# Predicting Adverse Drug Events in Chinese Pediatric Inpatients With the Associated Risk Factors: A Machine Learning Study

**DOI:** 10.3389/fphar.2021.659099

**Published:** 2021-04-27

**Authors:** Ze Yu, Huanhuan Ji, Jianwen Xiao, Ping Wei, Lin Song, Tingting Tang, Xin Hao, Jinyuan Zhang, Qiaona Qi, Yuchen Zhou, Fei Gao, Yuntao Jia

**Affiliations:** ^1^ Beijing Medicinovo Technology Co. Ltd., Beijing, China; ^2^ Ministry of Education Key Laboratory of Child Development and Disorders, Chongqing Key Laboratory of Pediatrics, Department of Pharmacy, National Clinical Research Center for Child Health and Disorders, Children’s Hospital of Chongqing Medical University, Chongqing, China; ^3^ Department of Hematology, Children’s Hospital of Chongqing Medical University, Chongqing, China; ^4^ Department of Ear-nose-throat, Children’s Hospital of Chongqing Medical University, Chongqing, China; ^5^ Department of Medical Record, Children’s Hospital of Chongqing Medical University, Chongqing, China; ^6^ Dalian Medicinovo Technology Co. Ltd., Dalian, China

**Keywords:** pediatric, machine learning, prediction, Chinese children, adverse drug event (s)

## Abstract

The aim of this study was to apply machine learning methods to deeply explore the risk factors associated with adverse drug events (ADEs) and predict the occurrence of ADEs in Chinese pediatric inpatients. Data of 1,746 patients aged between 28 days and 18 years (mean age = 3.84 years) were included in the study from January 1, 2013, to December 31, 2015, in the Children’s Hospital of Chongqing Medical University. There were 247 cases of ADE occurrence, of which the most common drugs inducing ADEs were antibacterials. Seven algorithms, including eXtreme Gradient Boosting (XGBoost), CatBoost, AdaBoost, LightGBM, Random Forest (RF), Gradient Boosting Decision Tree (GBDT), and TPOT, were used to select the important risk factors, and GBDT was chosen to establish the prediction model with the best predicting abilities (precision = 44%, recall = 25%, F1 = 31.88%). The GBDT model has better performance than Global Trigger Tools (GTTs) for ADE prediction (precision 44 vs. 13.3%). In addition, multiple risk factors were identified via GBDT, such as the number of trigger true (TT) (+), number of doses, BMI, number of drugs, number of admission, height, length of hospital stay, weight, age, and number of diagnoses. The influencing directions of the risk factors on ADEs were displayed through Shapley Additive exPlanations (SHAP). This study provides a novel method to accurately predict adverse drug events in Chinese pediatric inpatients with the associated risk factors, which may be applicable in clinical practice in the future.

## Introduction

Rising attention has been paid to the early warning of adverse drug events (ADEs) in hospitalized children. ADEs are defined as medication-related patient injury caused during any stage of the medication process, some of which are preventable due to errors, whereas some are adverse drug reactions (ADRs) and non-preventable (Desireé et al., 2009; Marcum et al., 2013; Malladi, 2016). The World Health Organization defines an ADR as a response to a noxious and unintended drug (Smyth et al., 2012). Events such as overdose, drug abuse, treatment failure, and drug administration errors are excluded from ADRs. In this study, we considered ADEs including ADRs and drug administration errors. ADEs can be manifested by signs, symptoms, or laboratory abnormalities, which are important causes of iatrogenic morbidity and mortality (Desireé et al., 2009).

As a special population, pediatric patients commonly have complicated situations, and the incidence of ADEs is hard to predict. A systematic review of 102 studies concluded that the incidence rates for ADRs causing pediatric admission ranged from 0.4 to 10.3% (Sakuma et al., 2014). Another study on Japanese pediatric inpatients found frequent ADEs with an incidence of 37.8 per 1,000 patient-days, and most were non-preventable (Morimoto et al., 2011). Surprisingly, the incidence of ADEs was around two times higher in admitted children than in adults (37.8 vs. 17.0 per 1,000 patient-days), and the incidence of medication errors was about eight times higher in admitted children than in adults (65.1 vs. 8.7 per 1,000 patient-days) (Poole, 2008). The possible reasons may be complexities in the pediatric medication process, which needs specific dosage calculation based on the age and weight of individual child; moreover, children are difficult to express and describe the symptoms of ADEs (Takata et al., 2008; Morimoto et al., 2011).

So far, the Global Trigger Tool (GTT), developed by the Institute for Healthcare Improvement (IHI), is a commonly used method for identifying potential ADEs among pediatric populations in the United States, the United Kingdom, Norway, Australia, and Japan (Grifn and Resear, 2009; Morimoto et al., 2011; Kirkendall et al., 2012; Chapman et al., 2014; Solevag and Nakstad, 2014; Hibbert et al., 2015; Ji et al., 2018). In China, Ji et al. explored the associated risk factors to predict ADEs using the GTT in children through stepwise logistic regression. The GTT uses “triggers” to identify ADEs, presenting as the ordering of certain medications, change of clinical status or symptoms, abnormal laboratory values, and abrupt stop orders (Resar et al., 2003; Marcum et al., 2013). However, based on previous research, pediatric patients have remarkable differences with regard to the risk factors associated with ADEs. Some found that gender, the number of drugs, use of antibacterial drugs, length of hospital stay, and general anesthesia were associated with ADEs in children. These findings still create controversy (Star et al., 2011; Rashed et al., 2012; Tiesen et al., 2013; Saedder et al., 2015; Andrade et al., 2017).

In our study, we aimed to apply machine learning methods to explore the associated risk factors for ADEs in Chinese pediatric inpatients. The rapidly developing machine learning methods can promote data-driven estimation when screening from multiple variables and capture nonlinear relations to achieve high accuracy in predicting clinical outcomes. We proposed to make a comparison between the study outcome and the findings of Ji et al., in order to find an optimal model to accurately predict pediatric ADEs and take effective prevention measures.

## Methods

### Study Design and Population

We enrolled pediatric inpatients from January 1, 2013, to December 31, 2015, in the Children’s Hospital of Chongqing Medical University, which is a large tertiary children’s hospital in China. Data were collected from the electronic medical records through the medical record system and the bar code system for medication administration. In order to compare the final results with those of the study by Ji et al., we applied the same criteria to select patients. The inclusion criteria were patients aged >28 days and <18 years, whose length of hospital stay >1 day and who were discharged or died between January 1, 2013, and December 31, 2015. The exclusion criteria were as follows: patients who had no drug exposure or were from the PICU, neonatal ward, hematology department, or oncology department (they were excluded because they had special treatment regimens that needed different triggers for ADE research). Samples were randomly selected from eligible patients using a random equidistant sampling method, obtaining a total of 1,800 patients. The whole dataset was then divided into derivation and test cohorts at the ratio of 8:2.

### Data Processing

Data were collected from medical records including patient’s basic information, diagnostic and treating procedures, medication charts, laboratory values, surgical records, nurse’s records, physician’s records, and admission and discharge records. One pharmacist and two pediatricians were assigned to examine the data and determine the occurrence of ADEs. If there was a disagreement, the final decision was made based on a consensus after team discussion. If the patient got actual harm that was related to medication, then the event was deemed as an ADE. Herein, harm was defined as an accidental body injury that needed medical care with additional monitoring, treatment, or hospitalization, including permanent injury or death. To be specific, the following symptoms or diseases were deemed as the occurrence of ADEs: gastrointestinal disorders (e.g., diarrhea, constipation, and vomiting), nervous system disorders (e.g., convulsions, convulsions grand mal, and over-sedation/hypotension), resistance mechanism disorders (e.g., candidiasis and fungal infection), metabolism and nutrition disorders (e.g., hyperkalemia, hypokalemia, hypoglycemia, hyperglycemia, and hyponatremia), respiratory system disorders (e.g., respiratory depression, bronchospasm, and dyspnea), rash, hepatotoxicity, nephritis, coagulopathy, leukopenia, allergic reactions, and so forth. The number of ADEs per case = the total number of ADEs/the number of cases.

### Selection of Risk Factors

Based on the data of pediatric inpatients’ records, the risk factors were screened from multiple patient characteristics. To be specific, we included patients’ demographic information (such as gender, age, weight, and height), status at birth (such as natural delivery/cesarean, premature birth, and weight at birth), information about admission (such as the number of medical diagnoses, admissions, admissions in the previous 1 year, and the length of hospital stay), and treatment information (such as surgical operation, number of drugs and doses, and the use of antibacterial, sedative analgesic, and anesthetic drugs). We set “the occurrence of ADEs” as the target variable to analyze which characteristic had remarkable influence on it. Subsequently, machine learning methods were applied to calculate the importance score of all risk factors according to patient characteristics, represented as a ranking figure. A factor with a higher risk score indicates more impact on the occurrence of ADEs. Based on the selected factors, we visually displayed the Shapley Additive exPlanations (SHAP) figure to demonstrate the positive or negative correlations between risk factors and the occurrence of ADEs (Lundberg and Lee, 2017).

### Model Establishment and Comparison

Using the selected risk factors as covariates, seven machine learning models were first established and analyzed through algorithms including eXtreme Gradient Boosting (XGBoost), CatBoost, AdaBoost, LightGBM, Random Forest (RF), Gradient Boosting Decision Tree (GBDT), and TPOT. The prediction metrics of the seven models were evaluated and compared in terms of the receiver operating characteristic (ROC) curve and the value of area under the curve (AUC), which represented the overall ability of classification and prediction. In order to compare the results with those of the study by Ji et al., precision/positive predictive value (PPV), recall, and F1 values of the prediction model were calculated. Precision/PPV indicates the number of times a risk factor independently identified an ADE divided by the number of times a risk factor was identified as positive. Ultimately, the algorithm with the best performance was selected to establish the model to predict the occurrence of ADEs in Chinese pediatric inpatients.

### Statistical Analysis

Data were analyzed by using Python 3.6.4 and WPS Office. Algorithms including XGBoost, CatBoost, AdaBoost, LightGBM, RF, GBDT, and TPOT were chosen to investigate risk factors associated with ADEs and the algorithm with the best performance was selected to establish the ADE prediction model. The evaluating metrics for model performance are as follows (Powers and Ailab, 2011):

$$\mathrm{Precision}=\frac{TP}{TP+FP},$$

$$\mathrm{Recall}=\frac{TP}{TP+FN},$$

F1=2×Precision×RecallPrecision+Recall.

TP, true positive, indicating the positive class is predicted as the number of positive classes; TN, true negative, indicating the negative class is predicted as the number of negative classes; FP, false positive, indicating the negative class is predicted as the number of positive classes; FN, false negative, indicating the positive class is predicted as the number of negative classes.

F1 is used to measure the merits and defects of the model, a larger F1 value indicating better model performance.

## Results

### Study Population

A total of 1,800 patients (cases) were enrolled in this study, while 54 patients were excluded, 28 of whom had no drug exposure and 26 of whom were diagnosed with cancer. The whole dataset was divided into derivation and test cohorts at the ratio of 8:2, which were 1,396 and 350 cases, respectively. According to Table 1, there is no significant difference between derivation and test cohorts (*p* > 0.05), except that gender and treatment with sedative analgesics have a slightly lower *p*-value (*p* = 0.02). In the final dataset of 1,746 cases, children were of the average age of 3.84 years, ranging from 0.08 to 17.75 years, females accounted for 35% (611 cases) and males 65% (1,135 cases), and the average body mass index (BMI) was 16.45 kg/cm^2^. The mean length of hospital stay was 7.83 days (ranging between 1 and 63 days), the average number of using drugs was 14 (1–64) per patient, and the average doses were 114 doses (1–1,206 doses) per patient. A total of 221 patients had ADEs, of which 32.6% were females, 77.4% were children with natural delivery, and proportions of children treated with antibacterial, sedative analgesic, and anesthetic drugs were 66.1, 43.0, and 52.5%, respectively. The relationships of these factors with the occurrence of ADEs need further screening in the following sections.

**TABLE 1:** Characteristics of patients with and without ADEs.

Variable	Derivation cohort (N = 1,396)	Test cohort (N = 350)	*p*-value	Total (n = 1,746)	Patients with ADEs (n = 221)	Patients with no ADEs (n = 1,525)
Demographics						
Female (%)	33.6%	40.6%	0.02	35.0%	32.6%	35.3%
Age (y)	3.8 ± 3.89	3.72 ± 3.89	0.35	3.84 ± 3.89	3.72 ± 4.12	3.86 ± 3.85
Weight (kg)	16.30 ± 11.66	15.54 ± 10.72	0.39	16.15 ± 11.48	15.37 ± 11.51	16.26 ± 11.47
Height (cm)	95.01 ± 29.37	93.46 ± 29.41	0.36	94.70 ± 29.38	91.97 ± 31.40	95.10 ± 29.06
BMI (kg/cm^2^)	16.49 ± 3.63	16.27 ± 3.13	0.84	16.45 ± 3.53	16.17 ± 2.85	16.49 ± 3.62
Developmental and nutritional status						
Fine	513 (36.7%)	126 (36.00%)	0.41	639 (36.6%)	70 (31.7%)	569 (37.3%)
Medium	757 (54.2%)	187 (53.43%)		944 (54.1%)	123 (55.7%)	821 (53.8%)
Lower middle	99 (7.1%)	25 (7.14%)		124 (7.1%)	19 (8.6%)	105 (6.9%)
Others	27 (1.9%)	12 (3.43%)		39 (2.2%)	9 (4.1%)	26 (2.0%)
Status at birth						
Natural delivery	382 (27.4%)	84 (24%)	0.22	1,280 (73.3%)	171 (77.4%)	1,110 (72.7%)
Cesarean	1,014 (72.6%)	266 (76%)		466 (26.7%)	50 (22.6%)	416 (27.3%)
Premature birth	63 (4.5%)	16 (4.57%)	0.92	79 (4.5%)	14 (6.3%)	65 (4.3%)
Weight at birth	3.22 ± 0.50	3.22 ± 0.51	0.83	3.22 ± 0.50	3.18 ± 0.49	3.22 ± 0.51
Admission						
Length of stay (d)	7.90 ± 5.51	7.58 ± 4.32	0.42	7.83 ± 5.29	10.23 ± 8.03	7.48 ± 4.66
Number of medical diagnoses	2.94 ± 1.89	3.11 ± 2.02	0.92	2.97 ± 1.89	2.92 ± 1.98	2.83 ± 1.71
Number of admissions	1.80 ± 1.43	1.88 ± 1.39	0.67	1.81 ± 1.42	2.07 ± 1.60	1.77 ± 1.39
Number of admissions in the previous 1 year	0.47 ± 1.02	0.59 ± 1.12	0.26	0.49 ± 1.04	0.61 ± 1.01	0.47 ± 1.04
Treatment						
Surgical operation	422 (30.2%)	90 (25.7%)	0.11	506 (29.0%)	64 (30.3%)	442 (28.8%)
Number of drugs	14.14 ± 6.82	14.31 ± 6.53	0.84	14.18 ± 6.77	18.82 ± 9.02	13.51 ± 6.01
Number of doses	114.24 ± 109.94	114.10 ± 87.77	0.83	113.94 ± 104.97	189.94 ± 187.00	102.92 ± 83.30
Antibacterial use	720 (51.6%)	194 (55.43%)	0.22	914 (52.3%)	146 (66.1%)	768 (50.4%)
Sedative analgesic use	587 (42.0%)	122 (34.86%)	0.02	709 (40.6%)	95 (43.0%)	614 (40.3%)
Anesthetic use	798 (57.2%)	199 (56.86%)	0.97	997 (57.1%)	117 (52.5%)	880 (57.7%)
Other						
Number of TT (+)	1.42 ± 1.49	1.56 ± 1.62	0.88	1.45 ± 1.51	2.95 ± 2.02	1.23 ± 1.29
ADEs	177 (79.1%)	44 (19.9%)	0.99	221 (12.7%)	221 (100%)	0

Abbreviations: BMI, body mass index; TT, trigger true; ADEs, adverse drug events

Notes: Data for variables are presented as mean ± variance, excluding those presented as cases and percentage (%). *p*-value is calculated for comparing the difference between derivation and test cohorts, *p* < 0.05 is considered significant.

### ADEs and Risk Factors

**FIGURE 1:**
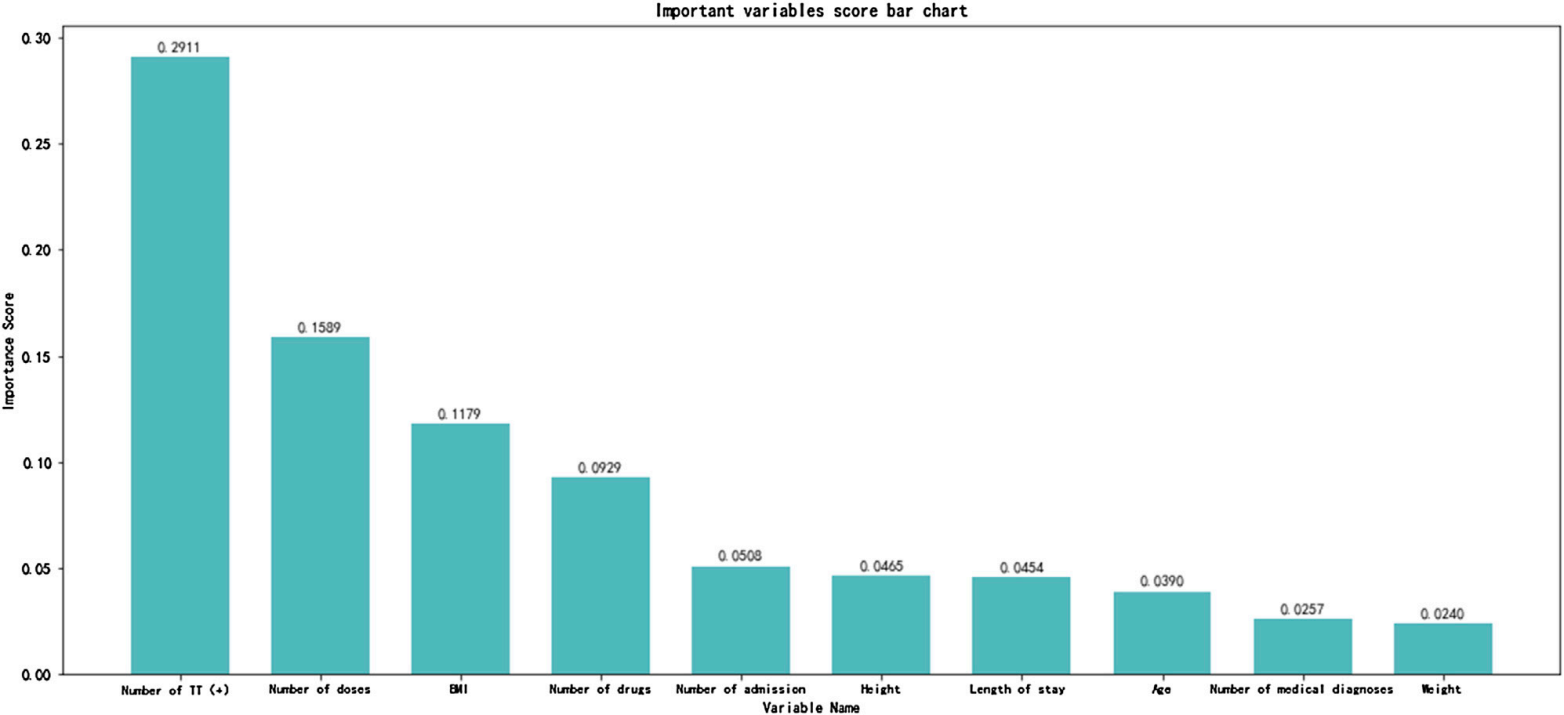
Importance score ranking for risk factors.

A total of 247 ADEs were identified in 221 patients, with an incidence rate of 12.7%. In Table 2, we summarize the classification of the drugs leading to the 247 ADEs. Anti-infective drugs including antibacterials, antivirals, and anti-tuberculosis drugs were the most common drugs causing ADEs in pediatric inpatients (35.9%). The importance scores of risk factors were calculated and ranked using seven algorithms. Since the GBDT model was ultimately proven to be the optimal one, Figure 1 only displays the importance score ranking in the GBDT model, the top 10 of which includes the number of trigger true [triggers were found to occur, TT (+)], number of doses, BMI, number of drugs, number of admission, height, length of hospital stay, weight, age, and number of diagnoses in a descending order. Among them, the number of TT (+) has the highest score of 0.2911, followed by the number of doses (0.1589) and BMI (0.1179), demonstrating their importance in predicting pediatric ADEs.

**TABLE 2:** Classification of drugs leading to occurrence of ADEs.

Classification of medicines	Types of medicines	Number of cases	Percentage (%)
Anti-infective drugs	Antibacterials	86	35.9
	Antivirals	3	
	Anti-tuberculosis drugs	1	
Nervous system drugs	Anti-epileptics	12	27.1
	Anti-anxiety drugs	1	
	Sedatives	55	
Digestive system drugs	Acid inhibitors	7	4.8
	Antidiarrheal drugs	5	
Hormonal and endocrine system drugs	Glucocorticoids	6	4.4
	Insulin	5	
Drugs to regulate water, electrolyte, and acid–base balance	Potassium chloride, glucose	10	4.0
Urological system drugs	Diuretics	4	2.0
	Dehydrating agent	1	
Antipyretic, analgesic, and anti-inflammatory drugs	Antipyretics	4	1.6
Cardiovascular medicines	Anti–heart failure drugs	1	1.2
	Anti-hypertensives	1	
	Anti-shock drugs	1	
Vitamins, minerals, amino acids, etc.	Minerals, amino acids	3	1.2
Hematology and hematopoietic system drugs	Anticoagulants	3	1.2
Anti-allergic reaction drugs	Anti-allergy drugs	2	0.8
Others	Immunomodulators	14	15.9
	Chinese herbal medicine/Chinese medicine injections	6	
	Mistake intake of paraquat, acetochlor, cocklebur	6	
	Blood products	5	
	Anesthetics	2	
	Medical tapes	1	
	Unspecified	6	

**FIGURE 2:**
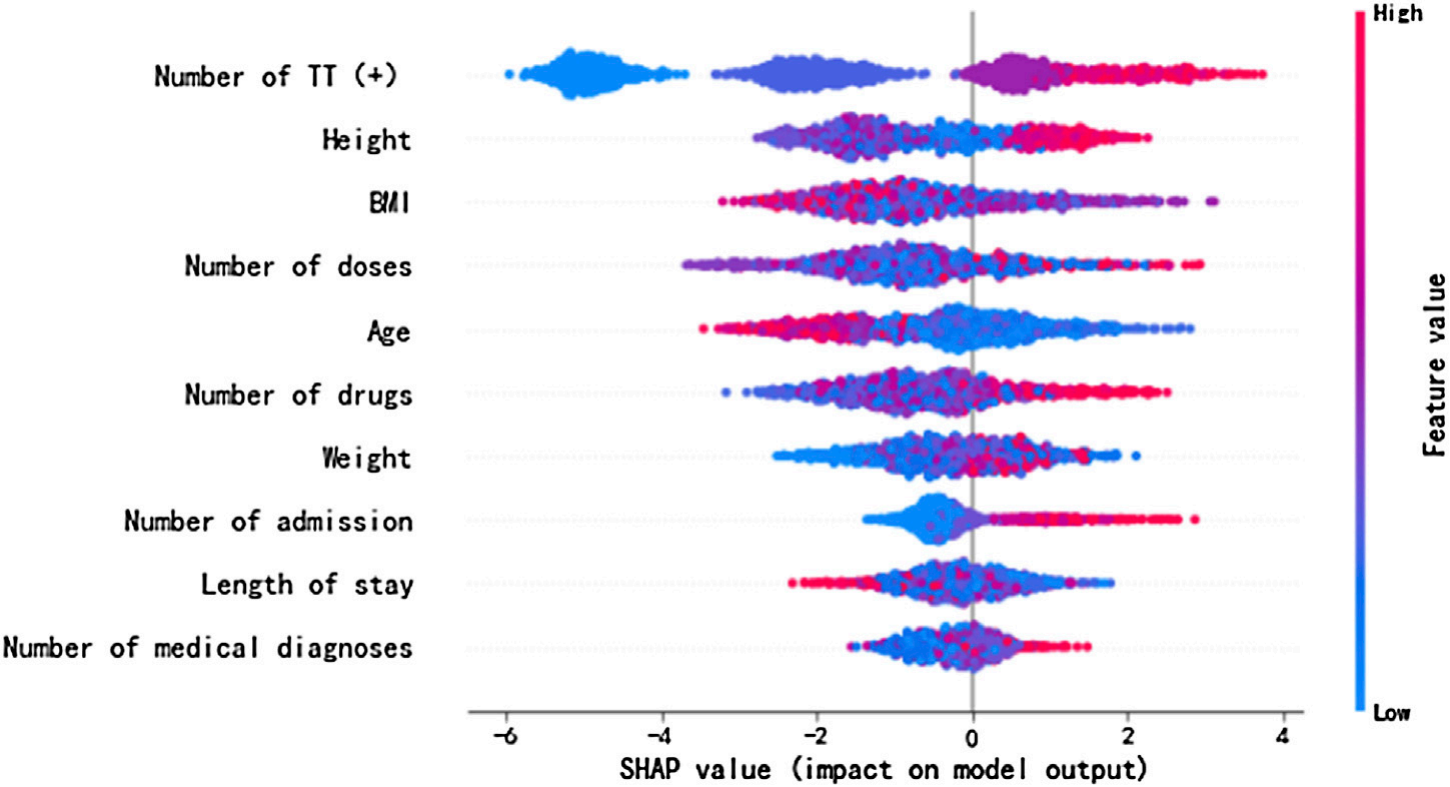
SHAP values of the important risk factors. The dot color is redder when the feature value gets higher and bluer when the feature value gets lower. When the SHAP value gets higher, the impact of the variable on model output is larger.

As depicted in Figure 2, for risk factors including the number of TT (+), number of doses, number of drugs, number of admission, number of diagnoses, and height, the dot color is redder when SHAP value gets larger and the color is bluer when SHAP value gets smaller, thus showing positive impacts of these factors on the risk of ADEs. Their SHAP values also show the same indications, which are 0.009, 0.082, 0.086, 0.011, 0.004, and 0.008 for the number of TT (+), number of doses, number of drugs, number of admission, number of diagnoses, and height, correspondingly. On the contrary, risk factors including age, BMI, and weight display negative impacts on the risk of ADEs, and their SHAP values are −0.003, −0.005, and −0.008, respectively. The length of hospital stay shows unclear direction of influence (SHAP = 0.001). Some display evident influencing directions, and others are relatively indistinct. With a larger sample size, the direction would be clear.

### Model Establishment and Comparison

**FIGURE 3:**
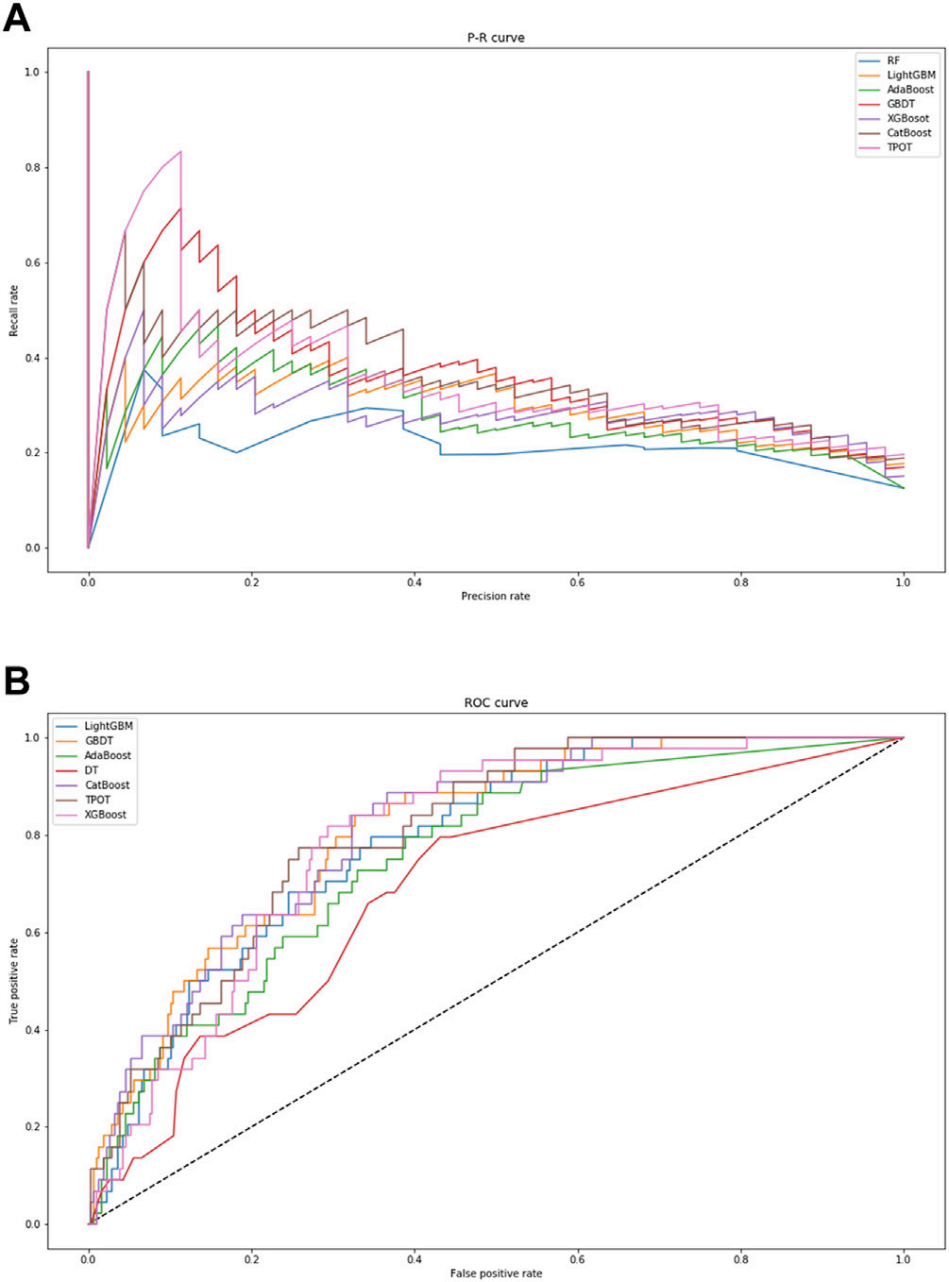
Visual presentation of model performance based on seven algorithms. **(A)** displays the precision–recall curve. **(B)** displays the ROC curve. When the area under curve is closer to “1,” the performance of model classification and prediction is better. Abbreviations: RF, Random Forest; GBDT, Gradient Boosting Decision Tree; XGBoost, eXtreme Gradient Boosting.

In Table 3, the metrics of seven models are compared in terms of precision, recall, and F1 value. Among the seven models, TPOT has the highest precision (75%) but moderate values of recall (13.64%) and F1 (23.08%), while GBDT has the highest values of recall (25%) and F1 (31.88%) with a moderate precision (44%). In addition, the visual comparisons of the seven models are displayed in Figure 3, including the precision-recall curve and the ROC curve, where the GBDT model achieves the highest AUC of 0.809. It can be seen that the GBDT model outperforms other models in the aspects of recall, F1, and AUC, demonstrating a good ability of model classification and prediction. After overall consideration of the predicting performance, we chose the model using the GBDT algorithm over the others to predict the occurrence of ADEs. Compared with the PPV of 13.3% in the study by Ji et al., the GBDT model has a precision of 44%, which surpassed their outcome (Marcum et al., 2013).

**TABLE 3:** Model performance using seven algorithms.

Model	Precision	Recall	F1
GBDT	44.00	25.00	31.88
LightGBM	27.27	6.82	10.91
AdaBoost	41.18	15.91	22.95
RF	23.08	13.64	17.14
CatBoost	46.15	13.64	21.05
TPOT	75.00	13.64	23.08
XGBoost	34.62	20.45	25.71

## Discussion

Prediction based on important risk factors is necessary for the prevention of ADEs in pediatric patients; nevertheless, it is difficult to achieve a precise prediction due to complex body status and dosing regimens of children. In the present study, we attempted to apply machine learning methods to deeply explore the risk factors associated with ADEs, since in the real-world studies, variables are not always independent of each other, and they are closely related in the nonlinear way. The normally used multivariate analysis methods cannot capture the complex relationships of variables, which machine learning methods are skilled in, especially GBDT that we used is able to divide and reaggregate variables to achieve the minimum prediction error when growing sub-trees. In this way, the nonlinear relationship between variables can be well captured. In addition, they all have the ability to learn from data with missing values directly, which can better adapt to the data situation in the real world. In the study by Ji et al., they found that an overall PPV of using trigger tools for ADE prediction was 13.3% at the Children’s Hospital of Chongqing Medical University, within the range of other trigger tools in pediatric care centers from 3.7 to 38% (Kirkendall et al., 2012; Marcum et al., 2013; Chapman et al., 2014; Unbeck et al., 2014; Solevag and Nakstad, 2014; Hibbert et al., 2015; Stockwell et al., 2015). In our study, the precision/PPV of the selected GBDT model was 44%, which outperforms the results of the study by Ji et al. and the majority of similar studies using trigger tools for ADE prediction.

Ji et al. found the significant risk factors for ADEs including the number of drugs, the number of doses, and the number of admissions (Marcum et al., 2013). Compared with their findings, our study identified the number of TT (+), BMI, height, weight, age, length of hospital stay, and number of drugs, doses, admission, and diagnoses, as the top 10 significant risk factors, which should be paid more attention on their measurement and take corresponding prevention in clinical. The trigger tools have proven their utility in multiple studies worldwide, some of which used IHI GTT (such as in the study by Ji et al., PPV 13.3%) and some of which developed other trigger tools, such as the U.S. pediatric-focused trigger tool (PPV 3.7%), the British National Health Service Pediatric Trigger Tool (PPV 19.8%), and the U.K. Pediatric Trigger Tool (Kirkendall et al., 2012; Marcum et al., 2013; Chapman et al., 2014; Solevag and Nakstad, 2014; Unbeck et al., 2014; Hibbert et al., 2015; Stockwell et al., 2015). Trigger tools show their practical ability in pediatric patients; however, the PPV of trigger tools was generally low and varied greatly among different populations and health care centers. We found that the number of TT (+) has a positive relationship with ADEs, which is also the most important risk factor, demonstrating that ADEs could be better predicted with more occurred triggers. Hence, it is highly recommended to increase the number of triggers and take them into consideration with other important risk factors together, in order to predict ADEs more accurately.

We also confirmed the importance of the number of drugs, doses, and admissions, which was consistent with the study by Ji et al. and previous research. The potential reason for the number of drugs as a risk factor could be the rising accumulated risks of multiple drug treatment, interactions between different drugs, and medication errors (Marcum et al., 2013). A similar reason can explain the number of doses being a risk factor, in that patients faced more risks of ADRs and the occurrence of overdose and drug abuse. As for the number of admissions, pediatric patients who were admitted frequently were commonly diagnosed with diseases requiring high-risk drugs, such as antiepileptic drugs for epilepsy, antibacterial drugs for recurrent infection, and some drugs for chronic diseases including corticosteroids, immunosuppressive agents, and analgesics (Rashed et al., 2012; Marcum et al., 2013). With regards to the number of diagnoses, a newly confirmed risk factor positively associated with ADEs in our study, generally, more drugs are used if the patient is diagnosed with more diseases. It can be explained by the increasing opportunities of drug–drug interactions, use of high-risk drugs, and occurrence of ADRs as well.

In terms of the hospital stay length, our result shows that it has an impact on the occurrence of ADEs. However, the length of hospital stay is commonly influenced by a couple of other factors, such as patient status, nursing care, and drug regimens (including the number of drugs and doses). Therefore, we did not consider the length of hospital stay as an independent risk factor for ADEs. In addition, some research believed that ADEs lead to prolonged length of hospital stay, which shows an inverse causal relationship (Rashed et al., 2012; Munoz-Torrero et al., 2010; Amelung et al., 2017). The causal relationship between length of hospital stay and ADEs is still a controversial topic currently, which needs further research in the future.

Of note, BMI, height, and weight were identified as remarkable risk factors. It is possibly because children have substantial variation in terms of weight and height, with their weights varying from 400 g to 120 kg (Takata et al., 2008). Moreover, most drugs need dosing calculation based on children’s weight, which may lead to a potential of 300-fold dosing errors (Takata et al., 2008). This is a noteworthy factor that needs careful records and strict reference of weight and height in order to predict pediatric ADEs in clinical practice. According to our findings, BMI and weight are negatively correlated to ADEs, indicating that children with low weight/BMI may experience more ADEs, possibly due to patient vulnerability as a result of low nutritional status.

Different from the findings of Ji et al., we found that age is a risk factor for the occurrence of ADEs, which was inconsistent with previous studies (Munoz-Torrero et al., 2010; Rashed et al., 2012). One indicated that age was not an independent risk factor of ADEs, as older patients showed more possibilities of having ADEs, which they believed was associated with more opportunities of using high-risk drugs (Rashed et al., 2012). In our viewpoint, younger children may be more vulnerable to ADEs because of the immature developmental and nutritional status and the susceptibility to drug reactions.

In conclusion, to our knowledge, this is a novel study to establish a prediction model for ADEs using machine learning in Chinese pediatric inpatients. The risk factors identified in this study could be incorporated into routine screen systems to improve inpatient safety in clinical practice. One drawback is the limited sample size, which needs to include more pediatric patient data in the future from different health care centers. Furthermore, the prediction model using GBDT should also be further validated in more pediatric inpatients including those in the hematology, oncology, PICU, and neonatal units.

## Data Availability

The data are available on request from the corresponding author.
